# Biological Activity of Naphthoquinones Derivatives in the Search of Anticancer Lead Compounds

**DOI:** 10.3390/toxins15050348

**Published:** 2023-05-20

**Authors:** Alexandra G. Durán, Nuria Chinchilla, Ana M. Simonet, M. Teresa Gutiérrez, Jorge Bolívar, Manuel M. Valdivia, José M. G. Molinillo, Francisco A. Macías

**Affiliations:** 1 Allelopathy Group, Department of Organic Chemistry, Institute of Biomolecules (INBIO), Campus de Excelencia Internacional (ceiA3), School of Science, University of Cadiz, 11510 Puerto Real, Cádiz, Spain; alexandra.garcia@uca.es (A.G.D.); nuria.chinchilla@uca.es (N.C.); ana.simonet@uca.es (A.M.S.); chema.gonzalez@uca.es (J.M.G.M.); 2 Department of Biomedicine, Biotechnology and Public Health-Biochemistry and Molecular Biology, Institute of Biomolecules (INBIO), University of Cádiz, República Saharaui 7, 11510 Puerto Real, Cádiz, Spain; mariateresa.gutierrez@gm.uca.es (M.T.G.); jorge.bolivar@uca.es (J.B.); manuel.valdivia@uca.es (M.M.V.)

**Keywords:** naphthoquinones, cytotoxic activity, juglone, lawsone, flow cytometry

## Abstract

Naphthoquinones are a valuable source of secondary metabolites that are well known for their dye properties since ancient times. A wide range of biological activities have been described highlighting their cytotoxic activity, gaining the attention of researchers in recent years. In addition, it is also worth mentioning that many anticancer drugs possess a naphthoquinone backbone in their structure. Considering this background, the work described herein reports the evaluation of the cytotoxicity of different acyl and alkyl derivatives from juglone and lawsone that showed the best activity results from a etiolated wheat coleoptile bioassay. This bioassay is rapid, highly sensitive to a wide spectrum of activities, and is a powerful tool for detecting biologically active natural products. A preliminary cell viability bioassay was performed on cervix carcinoma (HeLa) cells for 24 h. The most promising compounds were further tested for apoptosis on different tumoral (IGROV-1 and SK-MEL-28) and non-tumoral (HEK-293) cell lines by flow cytometry. Results reveal that derivatives from lawsone (particularly derivative **4**) were more cytotoxic on tumoral than in non-tumoral cells, showing similar results to those obtained with of etoposide, which is used as a positive control for apoptotic cell death. These findings encourage further studies on the development of new anticancer drugs for more directed therapies and reduced side effects with naphthoquinone skeleton.

## 1. Introduction

Cancer represents an important cause of morbidity and mortality worldwide, where 19.3 million new cases and 10 million cancer deaths were estimated in 2020. Additionally, a 47% increase in new cancer cases is expected in 2040 [1,2]. In Spain, this disease constitutes the second leading cause of death (25.2%) after those caused by diseases of the circulatory system (26.4%) in 2021 [3]. Nowadays, there is still a continuous effort in the search for more selective approaches and with reduced side effects to fight this illness.

Naphthoquinones have been known since ancient times due to their dye properties and their use in traditional medicine as wound-healing agents. Interest in these compounds has increased recently, owing to their broad range of biological activities, particularly their cytotoxic effects [4,5,6,7]. This cytotoxicity has been mainly ascribed to the ability of naphthoquinones to generate reactive oxygen species (ROS) as well as the electrophilicity of the quinone moiety to react with different biological targets through 1,4-Michael addition by the nucleophilic thiol species, proteins, DNA and RNA [8,9,10]. Other mechanisms of action have also been described, including the regulation of the tumor suppressor factor p53, inhibition of topoisomerase II, induction of apoptosis via ERS (endoplasmic reticulum stress) or Aurora-kinase inhibitors [11,12,13].

These natural products are widespread in nature and are characterized by possessing two carbonyl groups at positions 1,4 on the naphthalene ring, and more rarely at positions 1,2. Despite being isomers, these two different arrangements show different pharmacological actions due to their different physicochemical properties [14].

**Figure 1:**
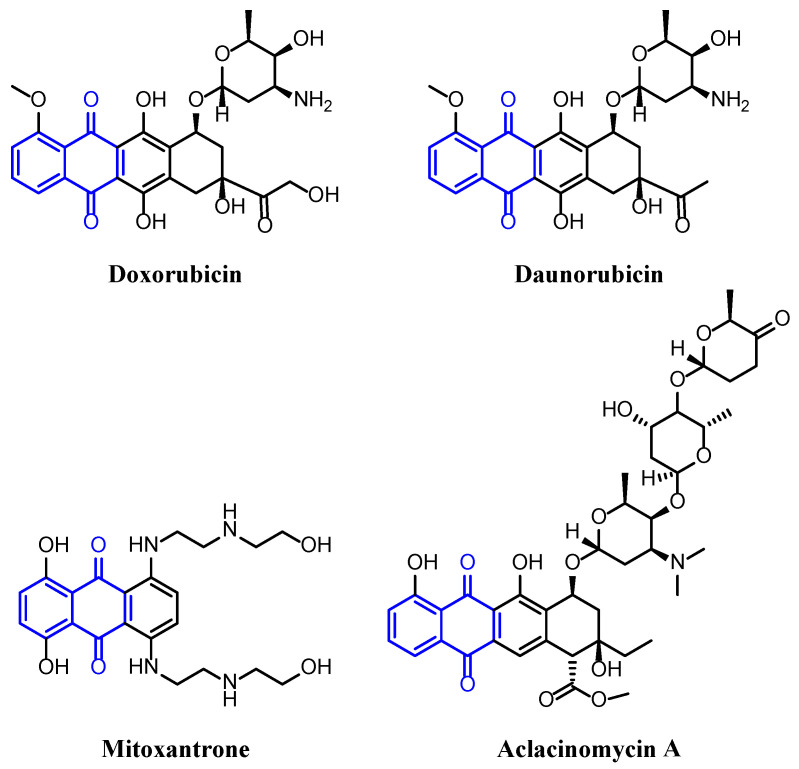
Examples of anticancer drugs that contain a 1,4-naphthoquinone skeleton in their structure.

Nowadays, many drugs based on natural products that contain a 1,4-naphthoquinone backbone in their chemical structure are used as anticancer agents, such as doxorubicin, daunorubicin, mitoxantrone and aclacinomycin A (Figure 1) [14,15,16]. In the search for new effective and selective chemotherapy approaches, naphthoquinones could play a crucial role.

In previous studies, a quantitative structure–activity relationship (QSAR) study was performed with two biologically active naphthoquinones, juglone (5-hydroxy-1,4-naphthoquinone (**1**)) and lawsone (2-hydroxy-1,4-naphthoquinone (**2**)). A correlation between the transport phenomena and the bioactivity of compounds was assessed to determine the optimal transport conditions [17]. These structures allow for easy chemical modifications that make themself suitable scaffolds for the study of their physicochemical properties in the search for more active compounds. A total of 44 synthesized *O*-acyl and *O*-alkyl derivatives were evaluated in a general activity bioassay named wheat coleoptile bioassay. This is an easy and rapid test (24 h) that is highly sensitive to a wide range of biological activities [18]. Different behaviors could be observed for each family of compounds, which are the modifications introduced at position 5 of the naphthoquinone backbone being more active.

The work described here concerns the evaluation of the cytotoxicity of the most active naphthoquinones derivatives from the previous wheat coleoptile bioassay study [17]. A preliminary cell viability bioassay was performed on cervix carcinoma (HeLa) cells for 24 h. The most promising compounds were further tested on different tumoral (ovarian carcinoma (IGROV-1) and human melanoma (SK-MEL-28)) and non-tumoral (human embryonic kidney 293 (HEK-293)) cell lines by flow cytometry.

## 2. Results and Discussion

**Figure 2:**
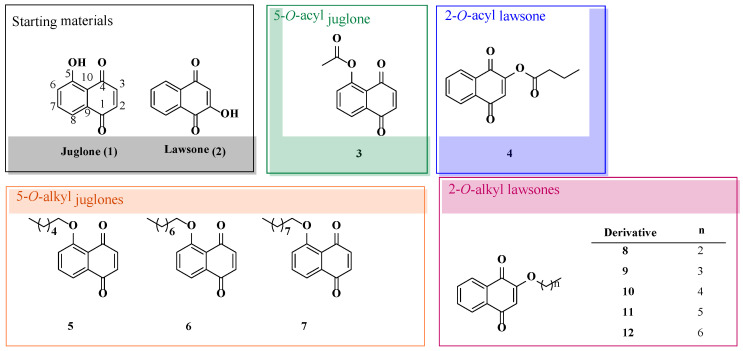
Naphthoquinones selected from the previous wheat coleoptile bioassay to perform the cytotoxicity study.

Ten naphthoquinones (**3**–**12**) selected from the wheat coleoptile bioassay (Figure 2) were evaluated in a first screening of cytotoxicity on HeLa cells at 100 μM for 24 h using the Trypan blue dye exclusion method. An untreated control (cells treated with 0.1% DMSO) and a positive control (cells treated with etoposide at 100 μM) were also included and evaluated for 24 h.

This is a simple, rapid, economic, and widely used method to assess cell viability after treatment with desired compounds [19]. Cells with damaged membranes are stained, while live cells with intact cell membranes are excluded and do not take up dye; however, this technique has two issues, and the results should be interpreted with caution. One of these concerns is that viability is being determined indirectly from cell membrane integrity, thus cell membrane integrity may be abnormal, yet the cell may be able to repair itself and become fully viable, but it would be determined as nonviable. On the other hand, dye uptake is assessed subjectively; small amounts of dye uptake indicative of cell injury may go unnoticed [20]. On the other hand, this method cannot explain the cause of cell death. Therefore, after completion of this first screening, the most active compounds were further evaluated by flow cytometry to detect apoptosis.

### 2.1. First Screening of Cytotoxicity on HeLa Cells by Trypan Blue Dye Exclusion Method

**Figure 3:**
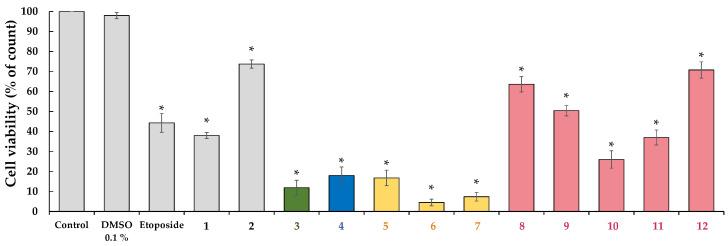
Cell viability by dye exclusion against cervical carcinoma cells (HeLa). Starting materials (**1** and **2**), products (**3**–**12**) classified by skeleton using different colors, and positive control (etoposide) were evaluated at 100 µM for 24 h. Experiments were performed in triplicate, and data are expressed as mean ± SD, *n* = 3, * *p* < 0.05 vs. untreated cells (DMSO 0.1%).

An *in vitro* cytotoxicity study on HeLa cells was used as a preliminary bioassay for screening of natural products with potential anticancer activity. Results of the ten naphthoquinones selected from the wheat coleoptile bioassay were assayed on HeLa cells for 24 h. are illustrated in Figure 3. These lead naphthoquinones are 5-acetoxy-1,4-naphthoquinone (**3**), 2-butanoyloxy-1,4-naphthoquinone (**4**), 5-*O*-alkyl juglones (chains of six, eight and nine carbon atoms, respectively) (**5**–**7**), and 2-*O*-alkyl lawsones (from three to seven carbon atoms) (**8**–**12**) (Figure 2). All the derivatives tested were more active than the corresponding starting materials, and most of them showed better growth inhibition than the positive control (etoposide), except for compounds **8**, **9** and **12**. It is also worth noting that an improvement of the activity could be observed for 2-*O*-alkyl lawsones with increase of side chain reaching the optimal value for derivative **10** (with 5 carbon atoms) and a loss of the activity with an increment in chain length. Taking into account each target backbone, the greatest activity values were noted for derivatives **3**, **4**, **6** and **10** with cell viability values lower than 30%.

### 2.2. Flow Cytometry Analysis of Cell Apoptosis on Ovarian Carcinoma (IGROV-1) Cells

Cancer is considered one of the main causes of morbidity and mortality in millions of people worldwide. Ovarian cancer is the seventh most common malignant tumor worldwide and the eighth cause of mortality in women [21]. Usually, most women are diagnosed with advanced stage cancer with a poor prognosis (with a 5-year survival rate of only 17% for a patient at an advanced stage), which is partly driven by delay in diagnosis and unequal access to quality care. Globally, around 428,000 new ovarian cancer cases and 307,000 deaths are predicted to occur in 2040 [22,23].

To date, one of the main drugs used in chemotherapy against this pathology is cisplatin. However, cisplatin resistance constitutes one of the main problems in antitumor therapy [24]. Despite the great therapeutic advances made in this area, there is not yet effective treatment that provides acceptable success rates and reduce the adverse effects. Therefore, there is still a pressing need to develop less harmful and cost-effective therapeutic alternatives; the discovery of novel anticancer drugs based in natural products is a key research field that has attracted a great interest in recent years.

**Figure 4:**
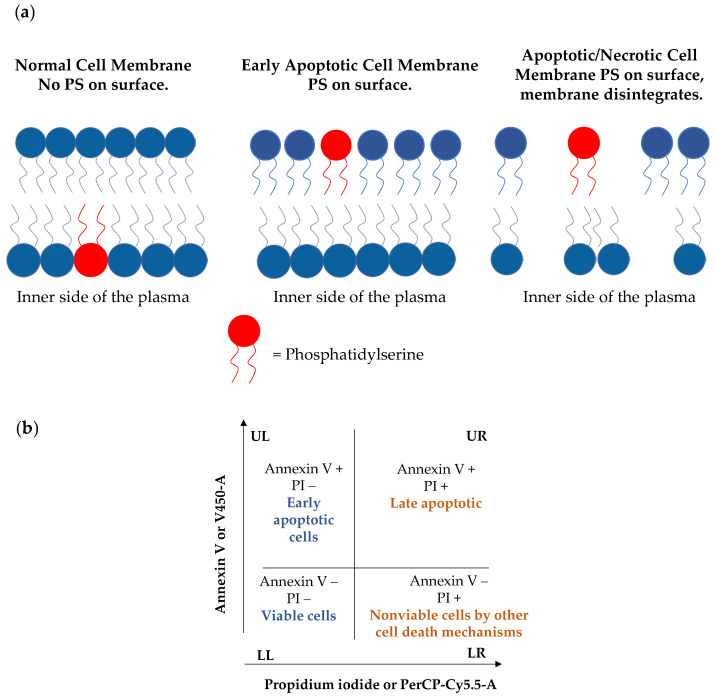
(**a**) Phosphatidylserine translocation and loss of membrane integrity in later stages of cell death; (**b**) plot illustrating flow cytometry analysis with AnnexinV-450 labelling apoptotic cells and 7-AAD (7-amino-actinomycin D) for cell death. Live cells are indicated in blue and death cells in orange.

Apoptosis, or programmed cell death, is characterized by numerous morphological and biochemical changes to the cellular architecture. It is well known that inappropriate apoptosis is implicated in many diseases, including neurodegenerative, ischemic, autoimmune disorders, and several forms of cancer [25]. One of the earlier events of apoptosis includes the translocation of membrane phosphatidylserine (PS) from the inner side of the plasma membrane to the surface, leading to the loss of plasma membrane asymmetry. On the other hand, PS translocation also precedes the loss of membrane integrity in later stages of cell death resulting from either apoptotic or necrotic processes (Figure 4a). Annexin V (a Ca^2+^-dependent phospholipid-binding protein) possesses high affinity for this phospholipid. Therefore, fluorochrome-labelled Annexin V can be used by flow cytometry in the detection of exposed phosphatidylserine at the outer leaflet of the plasma membrane, which is an indicator ofthe apoptotic death. Moreover, a vital dye, such as propidium iodide (PI), is typically used in conjunction with Annexin V for identification of early and late apoptotic cells. Viable cells with intact membranes exclude PI, whereas cell membranes or damaged cells are permeable to PI [26,27].

Therefore, a flow cytometric assay of Annexin V/PI was conducted to quantify the apoptotic profile in quinone-treated IGROV-1 cells. Naphthoquinones **3**, **4**, **6** and **10**, and etoposide (positive control) were tested at 100 µM for 24 h. Untreated cells (negative control) were also included in the experiment. These results are shown in dot plots, where the LL quadrant represents viable cells (both Annexin V and PI negative), the LR quadrant represents unviable cells (PI positive and Annexin V negative), the UL quadrant represents cells in early apoptosis/cell apoptosis (Annexin V positive and PI negative) and UR quadrant represents cells that are in late apoptosis or necrosis (both Annexin V and PI positive) (Figure 4b).

**Figure 5:**
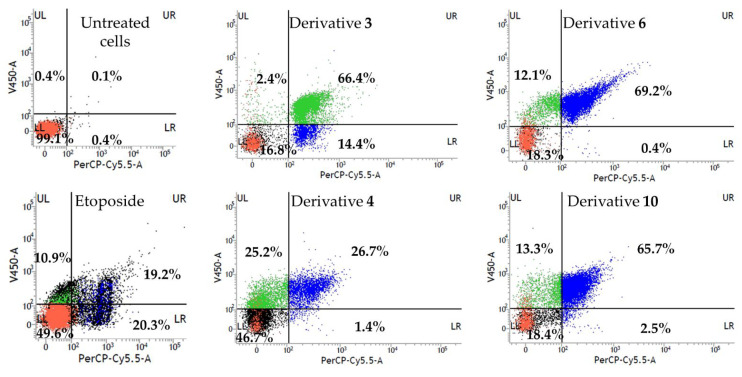
Flow cytometry results for the derivatives **3**, **4**, **6** and **10** on IGROV-1 cells at 100 µM for 24 h. Etoposide was used as positive control.

In the negative control (untreated cells), most of the cells were viable (99.0%) and showed to be non-apoptotic. In contrast, significant differences could be observed after the treatment of the naphthoquinones (Figure 5). A remarkable increase in apoptotic cell number (UL + UR quadrants) was observed compared to the untreated cells. It is worth highlighting that derivatives **4**, **6**, and **10** produced a significant increase in early and late apoptotic cells (52−81%) while the population of nonviable cells by other death mechanisms scarcely increased (0.4−2.5%). Moreover, derivative **3** caused an increase in apoptotic or dead cells from untreated to treated cells (0.1% to 66.4%, respectively), together with a lesser increase in the early apoptotic cell populations (2.4%) with only 16.8% of viable cells.

**Figure 6:**
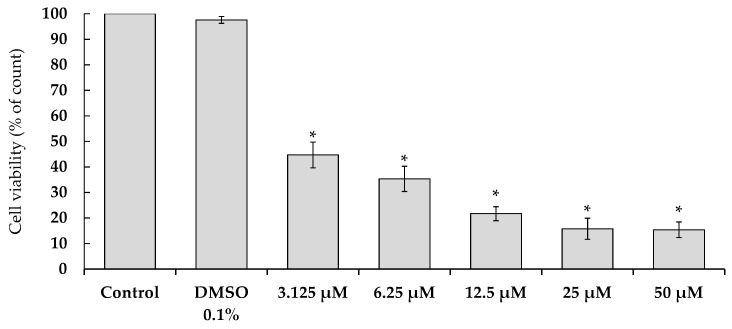
Cell viability by dye exclusion against ovarian carcinoma cells (IGROV-1) of compound **3** from 3.125 to 50 µM for 24 h. Etoposide and untreated cells (0.1% DMSO) were used as a positive and negative control, respectively. Experiments were performed in triplicate, and data are expressed as mean ± SD, *n* = 3, * *p* < 0.05 vs. untreated cells (DMSO 0.1%).

In view of the results outlined above, derivative **3** was selected to perform a time course study over various concentrations in IGROV-1 cell line by cell viability assay to find the optimal conditions for its evaluation by flow cytometry in order to know if the early apoptotic cell population would increase. A concentration range from 3.125 to 50 µM and a time range of 3–48 h were evaluated. Results indicated that compound **3** inhibited the IGROV-1 cell growth after 24 h of incubation in a dose-dependent manner (Figure 6).

**Figure 7:**
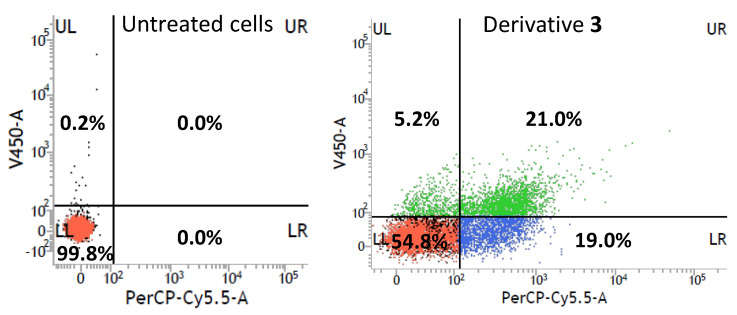
Flow cytometry results for the derivative **3** on IGROV-1 cells at 3.125 µM for 24 h. Apoptosis was quantitatively assessed after cells were stained with Annexin V-450 and 7-AAD.

Two concentrations were selected from the study over time (3.125 and 6.25 µM), which showed values of 55.3 and 64.7% of growth inhibition, respectively, in ovarian carcinoma cells for 24 h. Results obtained with these concentrations in a time range of 3–48 h are illustrated in Figures S1 and S2. A 46.4% growth inhibition was reached at 3.125 µM for 24 h, therefore these conditions were considered optimal for their study by flow cytometry due to the less pronounced decrease in cell viability over time. Flow cytometry analysis under these optimal conditions for derivative **3** is shown in Figure 7. In this case, the number of viable cells increased to 54.8% in comparison to the previous experiment with a 16.8% of viable cells at 100 µM. Therefore, the drastic conditions and high cell death decreased. Moreover, a 5.2% of early apoptotic cells, 21% of apoptotic cells and 19% of nonviable cells were observed. Although an increase in the number of viable cells under these conditions (3.125 µM and 24 h) had been achieved, the number of early apoptotic cells was similar to that of 100 µM and 24 h (5.2% and 2.4%, respectively), and even the number of nonviable cells by other cell death mechanisms was higher in this last case with the optimal conditions (19.0%).

Nevertheless, taking into account the results obtained for the other three naphthoquinone derivatives (**4**, **6**, and **10**), it is worth mentioning that the number of nonviable cells by other cell mechanisms different to apoptosis is very small (0.4–2.5%), and the number of early and late apoptotic cells was increased (52–81%), making apoptosis the primary cell death mechanism. These findings propose these structures as candidates to find more oriented therapies. To gain further insight into the apoptosis cell death, these naphthoquinones were further evaluated on human melanoma cells (SK-MEL-28) and non-tumoral human embryonic kidney 293 (HEK-293) cells.

### 2.3. Flow Cytometry Analysis of Cell Apoptosis on Human Melanoma (SK-MEL-28) and Non-Tumoral Human Embryonic Kidney 293 (HEK-293) Cells

**Figure 8:**
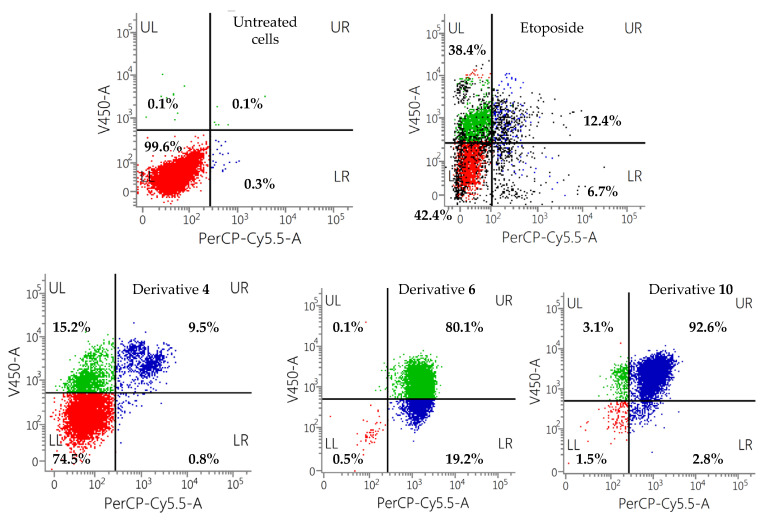
Flow cytometry results for the derivatives **4**, **6**, and **10** on SK-MEL-28 cells at 100 µM for 24 h. Etoposide was used as positive control.

**Figure 9:**
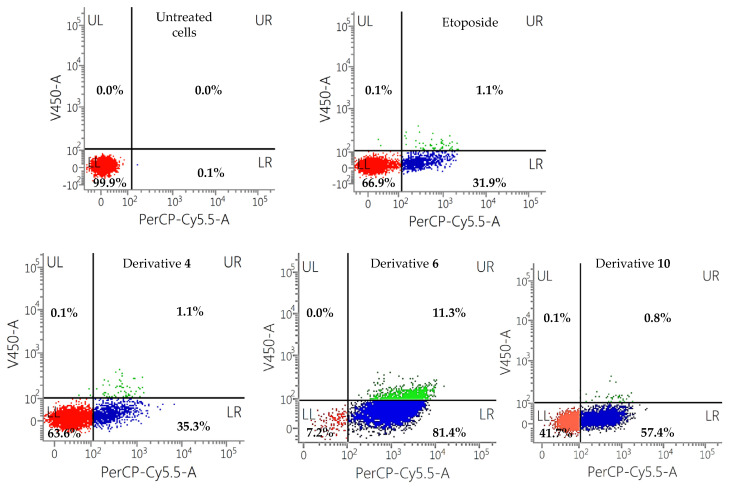
Flow cytometry results for the derivatives **4**, **6**, and **10** on HEK-293 cells at 100 µM for 24 h. Etoposide was used as positive control.

Derivatives with the most promising results (2-butanoyloxy-1,4-naphthoquinone (**4**); 5-octoxy-1,4-naphthoquinone (**6**); and 2-pentoxy-1,4-naphthoquinone (**10**)) that trigger cell death by apoptotic processes were evaluated on a different tumoral (human melanoma) and non-tumoral (human embryonic kidney 293) cells (Figure 8 and Figure 9).

The overall prevalence and incidence of both non-melanoma (basal cell and squamous cell carcinomas) and melanoma skin cancers have increased over the past decades. It is estimated that between 2 and 3 million non-melanoma skin cancers and 132,000 melanoma skin cancers occur globally each year. Surgical excision of these malignancies remains the traditional mainstay of treatment as well as topical treatments with semisolid formulations (with 5-fluorouracil, diclofenac, and imiquimod) and photodynamic therapy, among others. Despite the remarkable progress in cancer treatment over the last decades, the survival rate of many patients remains slow. Additionally, owing to the difficulties in clinical trials, there is no FDA (Food and Drug Administration) registered topical treatments of skin cancer so far [28,29,30,31].

The human embryonic kidney 293 (HEK 293) cell line was developed in 1977 from the sheared Adenovirus 5 (Ad5) DNA transformation of the human embryonic kidney cell [32]. HEK-293 cells are relatively easy to transfect and widely used to study a large variety of biological processes [33]. These cells are considered one among the widely used standards for non-tumoral human cells [34]. For these reasons, the HEK-293 cell line was chosen to gain better insights into the selectivity of the lead compound against tumoral and non-tumoral cells [35].

In view of the results obtained, it can be drawn that the most cytotoxic naphthoquinone derivative in all the cell lines tested is the derivative 5-*O*-acyl juglone (**6**), with a percentage of viable cells of 18.3%, 0.5%, and 7.2% on IGROV-1, SK-MEL-28, and HEK-293 cells, respectively. On the other hand, naphthoquinones modified at position 2 of the naphthoquinone backbone (2-*O*-acyl lawsone (**4**) and 2-*O*-alkyl lawsone (**10**), showed certain selectivity among the different cell lines assayed.

2-butanoyloxy-1,4-naphthoquinone (**4**) showed significant cytotoxic activity on ovarian carcinoma cells, with 51.9% of apoptotic cells (UL + UR quadrants) and 46.7% of viable cells, showing better results than the positive control etoposide (30.1% of apoptotic cells and 49.6% of viable cells). Nevertheless, it did not show a significant cytotoxic activity on melanoma cells, with 74.5% of viable cells and 24.7% of apoptotic cells. It is worth highlighting that 63.6% of viable cells were observed on non-tumoral HEK-293 cells, with similar cytotoxic values showed by the positive control (66.9% of viable cells). These results highlight the higher toxicity on IGROV-1 than in SK-MEL-28 tumoral cells and in non-tumoral human embryonic kidney cells (HEK-293) at 100 µM for 24 h.

On the other hand, derivative **10** (2-pentoxy-1,4-naphthoquinone) showed high cytotoxicity in the two tumoral cell lines tested, reaching activity values better than etoposide (18.4% of viable cells and 79% of apoptotic cells on IGROV-1 and 1.5% of viable cells and 95.2% of apoptotic cells on SK-MEL-28 cells). Regarding the cytotoxicity on non-tumoral cells, it showed 41.7% of viable cells.

It has been demonstrated that different substitution patterns on the naphthoquinone moiety, as well as the position of the hydroxyl groups, may play a crucial role in the activity observed, since it affects the redox potentials and pro-oxidant activities [36]. Thus, there are several studies that describe this fact. Ali and co-workers evaluated the effect of antileishmanial activity of a series of naphthoquinones, including simple, oxygenated in the aromatic or quinonoid ring, dimeric and furanonaphthoquinones. Leishmanicidal activity was strongly dependent on the nature and position of these substituents. The most active compounds were those with hydroxylation at C-5 and dihydroxy substitution at C-5 and C-8 on the napththoquinone ring. In contrast, 2-hydroxynaphthoquinones were less active [36]. Another study performed by Wang and co-workers, describes the preparation of a series of 2-position and 3-position lipophilic-substituted lawsone and juglone derivatives to evaluate their anticancer activity in vitro. Most of the more active compounds with better activity values were those with 2-*O*-alkyl-, or 3-*C*-alkyl- derivatives synthesized from lawsone, which are 2-hydroxy-3-farnesyl-1,4-naphthoquinone, the most cytotoxic compound against the cell lines tested [4]. Likewise, a SAR study of the cytotoxicity of a series of 1,4-naphthoquinones was performed by Shen and co-workers, where the high cytotoxicity was observed for juglone derivatives [37].

Moreover, one of the main disadvantages described for this kind of compounds is its high cytotoxicity and its low therapeutic selectivity. Nevertheless, these results reveal certain selectivity, especially for derivative **4** with strong cytotoxicity on ovarian carcinoma cells and non-significant toxicity on non-tumoral HEK-293 cells, with values similar to the positive control etoposide. Furthermore, it was noted that modifications performed at position 2 of the naphthoquinone backbone were more cytotoxic on tumoral cells than in non-tumoral (2-*O*-acyl and 2-*O*-alkyl derivatives) in comparison to 5-*O*-acyl and 5-*O*-alkyl derivatives.

The starting materials, juglone (found in leaves, roots, husks, and barks of several species of walnut trees) and lawsone (isolated from the leaves and shoots of henna, *Lawsonia inermis* L.) have shown cytotoxicity against a wide range of human cancer cell lines [38,39,40,41]. Regarding the analogues investigated, cytotoxic properties for derivatives **4**, **6** and **10**, are described for the first time. In contrast, the cytotoxic properties of derivative **3** (5-acetoxy-1,4-naphthoquinone) have been previously reported in the literature. It has shown strong cytotoxicity on human oral epidermoid carcinoma (KB) cell line with an IC_50_ value of 1.39 µM [37], and it has also shown cytotoxicity against L-60 (leukemia), MDA-MB-435 (melanoma), SF-295 (brain) and HCT-8 (colon), human cancer cell lines [41]. In our experiments, an IC_50_ value of 7.54 µM for derivative **3** was obtained for ovarian carcinoma (IGROV-1) cells.

## 3. Conclusions

Naphthoquinones have been known for a long time to show cytotoxic properties [4]. These effects are mainly ascribed to the ability of these scaffolds to generate ROS and also to react with different biological targets, such as DNA topoisomerase [42]. However, they have been considered as PAINS (pan-assay interference compounds) in high-throughput screenings and are therefore considered as notorious troublemakers by the scientific community [14]. Nevertheless, these kind of compounds have been used for many medicinal traditional uses since ancient times and a great number of approved drugs or that are in preclinical trials even possess a naphthoquinone skeleton. Moreover, these compounds possess multitarget activity, which can be employed as synergic polypharmacology in chemotherapies treatments and also to fight resistant tumoral cells. It has been previously reported that a large number of naphthoquinones have shown high efficacy in tumoral cell lines resistant to drugs or chemotherapeutic agents [14,43,44].

A series of 1,4-naphthoquinone derivatives have been evaluated on different tumoral (HeLa, IGROV-1, and SK-MEL-28) and non-tumoral (HEK-293) cell lines. Results denoted that derivatives from lawsone showed more selectivity against tumoral cells, while their cytotoxicity on non-tumoral cells were similar to the positive control etoposide. On the other hand, those derivatives from juglone result to be more cytotoxic against all the cell lines tested. Therefore, the number of apoptotic cells was quantified and assayed by flow cytometry. Derivatives **4** (2-butanoyloxy-1,4-naphthoquinone) and **10** (2-pentoxy-1,4-naphthoquinone) cause apoptosis to be the primarily cell death mechanism. These findings encourage further studies on the development of new anticancer drugs with a naphthoquinone backbone, with improved selectivity, avoiding the induction of drug resistance and reduce toxicity. Cytotoxicity of derivatives **4**, **6**, and **10** is described for the first time.

## 4. Materials and Methods

### 4.1. Chemicals and General Experimental Procedures

5-Hydroxy-1,4-naphthoquinone (juglone, technical grade, 97%) and 2-hydroxy-1,4-naphthoquinone (lawsone, 98%) were supplied by Alfa Aesar (Heysham, UK) and Acros Organics (Morris Plains, NJ, USA), respectively.

Dimethyl sulfoxide was supplied by Panreac Quimica SAU (Castellar del Vallés, Barcelona, Spain). Dulbecco’s Modified Eagle’s Medium (DMEM) was supplied by Lonza (Verviers, Belgium), premixed phosphate-buffered saline solution (PBS, 10×) was supplied by Roche (Steinheim, Germany), fetal bovine serum, penicillin/streptomycin, L-glutamine, sodium pyruvate, trypsin, and minimum essential medium non-essential amino acids (MEM NEAA), were purchased from Gibco (Paisley, UK). BD Annexin V: FITC Apoptosis Detection Kit I was supplied by BD Biosciences (Madrid, Spain).

### 4.2. Cell lines and Cell Cultures

HeLa (human cervix carcinoma), IGROV-1 (human ovarian carcinoma), SK-MEL-28 (human melanoma), and HEK-293 (human embryonic kidney 293) cells were cultured as monolayers in DMEM (GIBCO) supplemented with 10% fetal bovine serum, 5% glutamine, 5% non-essential amino acids, 5% penicillin–streptomycin, and 5% sodium pyruvate. Cells were maintained in a Hera Cell 150i (Thermo Scientific, Waltham, MA, USA) incubator at 37 °C, 5% CO_2_ and 95% humidity. For all assays, cells were allowed to attach until 70% confluence was reached prior to treatment.

### 4.3. Cell Viability Assays

The different cell lines were sterile cultured in 6-well plates (VWR, Darmstadt, Germany) each to 70% confluence during at least 48 h. Cell lines were treated for 24 h with the corresponding naphthoquinone derivative in DMSO (0.1% *v/v*) at a concentration of 100 µM. In addition, concentration ranges from 3.125 µM to 50 µM and treatments ranging from 3 h to 48 h of product **3** were also evaluated. Control cultures, including cells treated with 0.1% DMSO and positive control of cells treated with etoposide at 100 μM, were also included in each experiment.

Trypan blue solution (0.4% Sigma Aldrich, Steinheim, Germany) was mixed 1:1 with a sample of control or treated cells. After incubation for 2 min, a fraction of blue-stained cells was assessed using an Automated Cell Counter T20 (Bio-Rad, Hercules, CA, USA). Experiments were performed in triplicate; data are expressed as the mean of triplicate measurements (mean ± SD).

% viability = (Total number of viable cells/Total number of viable and non-viable cells) × 100

### 4.4. Flow Cytometry and Data Analysis

Detection of apoptosis was performed using the kit Annexin V FITC assay, which contains Annexin V-FITC, propidium iodide staining solution, and Annexin V binding buffer.

The fluorescence of single cells was measured by a FACSVerse^TM^ bench top flow cytometer equipped with blue (488 nm), red (640 nm) and violet (405 nm) lasers. Amplification of signals were carried out at logarithmic scale and measurements of events were plotted on forward light scatter (FSC), side light scatter (SSC), violet fluorescence to detect Annexin-V 450 and red fluorescence for 7-AAD. A gating strategy was used to distinguish the fluorescently labelled cell population from unstained populations. A total of 10,000 events as defined by gates were counted and cell counts were expressed as percentage. The data were analyzed using BD Assurity Linc^TM^ Software v 1.0.

After treatment with the products, cell culture medium was collected into 15 mL tubes, and each well was cleaned with PBS (1 mL). Accutase was added to each well, enough to cover the surface (300 µL) and incubated for 1–2 min at room temperature. Then, 2 mL of PBS was added to each well, and the contents transferred to the 15 mL tubes. They were centrifuged, and the supernatant was discarded. Cells were transferred to an eppendorf and washed with cold PBS in triplicate. They were resuspended in 1×X Binding buffer (100 µL), and then Annexin V-450 (5 µL) and 7-AAD (7-amino-actinomycin D, 2.9 µL) were added. They were diluted again with 400 µL of 1× binding buffer, and the cells were mixed and incubated for 15 min at room temperature in the dark prior to analysis. Measurements were done within 1 h.

### 4.5. Statistical Analysis

The results are presented as the mean ± standard deviation (SD) of at least three independent experiments. Data were evaluated with GraphPad Prism^®^ Version 5.00 software (San Diego, CA, USA) and analyzed using one-way ANOVA. Values were considered to be statistically significant when *p* < 0.05. Additionally, an IC_50_ value for derivative **3** was determined using the same software.

## Data Availability

Not applicable.

## Supplementary Materials

The following supporting information can be downloaded at: https://www.mdpi.com/article/10.3390/toxins15050348/s1, Figure S1: Cell viability by dye exclusion of derivative **3** on ovarian carcinoma cells (IGROV-1) at 3.125 µM; Figure S2: Cell viability by dye exclusion of derivative **3** on ovarian carcinoma cells (IGROV-1) at 6.25 µM.
