# Supplementary material for: Biological Activity of Naphthoquinones Derivatives in the Search of Anticancer Lead Compounds
Source: Toxins (Basel). 2023 May 20;15(5):348. doi: 10.3390/toxins15050348 (PMC10222971; doi:10.3390/toxins15050348)
Supplement: Supplementary file 1 [file toxins-15-00348-s001.zip › toxins-2394961-supplementary.pdf]

toxins

MDPI

Supplementary Materials: Biological Activity of Naphthoquinones Derivatives in the Search of Anticancer Lead Compounds

Alexandra G. Durán, Nuria Chinchilla, Ana M. Simonet, María Teresa Gutiérrez, Jorge Bolívar, Manuel M. Valdivia, José M. G. Molinillo and Francisco A. Macías

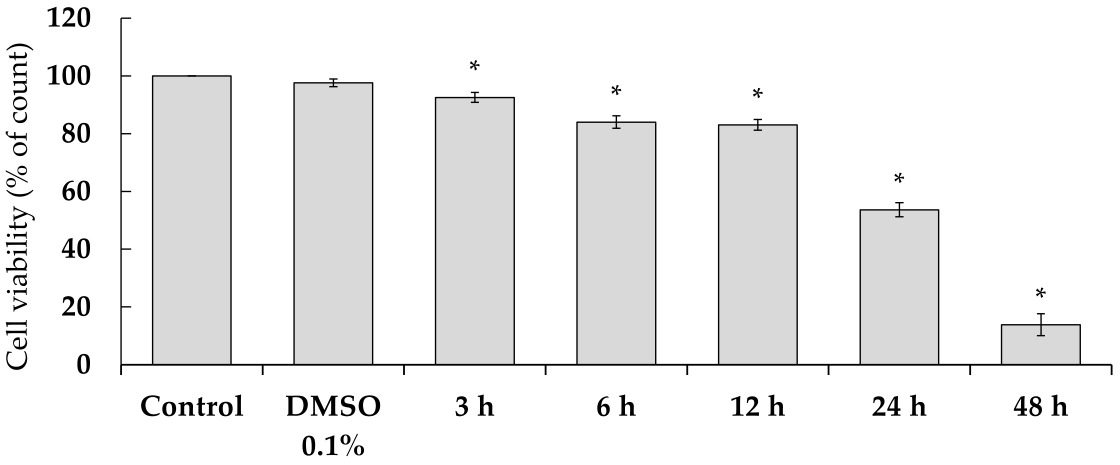

Figure S1. Cell viability by dye exclusion of derivative **3** on ovarian carcinoma cells (IGROV-1) at 3.125 μM. Experiments were performed in triplicate and data are expressed as mean ± SD, $n = 3$, * $p < 0.05$ vs. untreated cells (DMSO 0.1%).

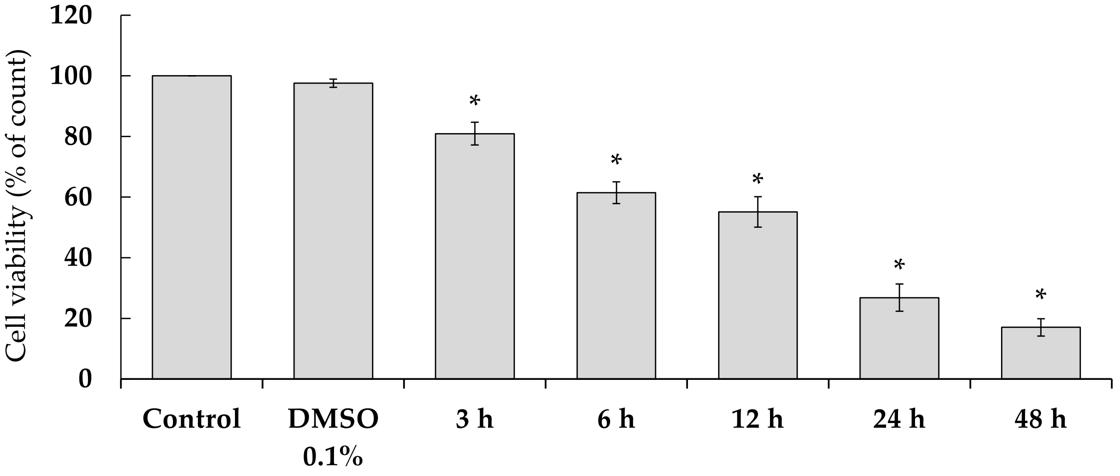

Figure S2. Cell viability by dye exclusion of derivative **3** on ovarian carcinoma cells (IGROV-1) at 6.25 μM. Experiments were performed in triplicate and data are expressed as mean ± SD, $n = 3$, * $p < 0.05$ vs. untreated cells (DMSO 0.1%).
